# 3D-QSAR and docking studies of flavonoids as potent *Escherichia coli* inhibitors

**DOI:** 10.1038/srep23634

**Published:** 2016-04-06

**Authors:** Yajing Fang, Yulin Lu, Xixi Zang, Ting Wu, XiaoJuan Qi, Siyi Pan, Xiaoyun Xu

**Affiliations:** 1 Key Laboratory of Environment Correlative Dietology (Huazhong Agricultural University), Ministry of Education, Wuhan 430070, P.R. China; 2 Agricultural Bioinformatics Key Laboratory of Hubei Province, College of Informatics, Huazhong Agricultural University, Wuhan 430070, P.R. China; 3 Oilcrops Research Institute, Chinese Academy of Agricultural Sciences, Wuhan 430062, P.R. China; 4 College of Science, Huazhong Agricultural University, Wuhan 430070, P.R. China

## Abstract

Flavonoids are potential antibacterial agents. However, key substituents and mechanism for their antibacterial activity have not been fully investigated. The quantitative structure-activity relationship (QSAR) and molecular docking of flavonoids relating to potent anti-*Escherichia coli* agents were investigated. Comparative molecular field analysis (CoMFA) and comparative molecular similarity indices analysis (CoMSIA) were developed by using the pIC_50_ values of flavonoids. The cross-validated coefficient (*q*^*2*^) values for CoMFA (0.743) and for CoMSIA (0.708) were achieved, illustrating high predictive capabilities. Selected descriptors for the CoMFA model were ClogP (logarithm of the octanol/water partition coefficient), steric and electrostatic fields, while, ClogP, electrostatic and hydrogen bond donor fields were used for the CoMSIA model. Molecular docking results confirmed that half of the tested flavonoids inhibited DNA gyrase B (GyrB) by interacting with adenosine-triphosphate (ATP) pocket in a same orientation. Polymethoxyl flavones, flavonoid glycosides, isoflavonoids changed their orientation, resulting in a decrease of inhibitory activity. Moreover, docking results showed that 3-hydroxyl, 5-hydroxyl, 7-hydroxyl and 4-carbonyl groups were found to be crucial active substituents of flavonoids by interacting with key residues of GyrB, which were in agreement with the QSAR study results. These results provide valuable information for structure requirements of flavonoids as antibacterial agents.

The abuse of synthetic antibiotics has contributed to the increased incidence of bacterial resistance to available antibacterial agents, resulting in an urgent need for natural antimicrobials^1^. Flavonoids, natural compounds widely distributed in edible plants, are well documented for their antibacterial activities^2^,^3^,^4^. Structurally, most flavonoids share a diphenylpropane (C6-C3-C6) parent skeleton. Depending on the structure difference on the C ring, flavonoids are mainly divided into eight subclasses including flavones, flavonols, flavanones, flavanonols, isoflavones, anthocyanins, chalcones and flavan-3-ols^5^. The antibacterial activities of flavonoids have been reported to be related to their chemical structures^6^. The number of substituents and positions such as hydroxylation^7^, methoxylation^8^, prenyl groups or glycosylation^9^ are related to differences in antibacterial activities. Structure activity relationships (SAR) of flavonoids have been obtained by comparing the activities data and structure difference of two analogues. The lack of prediction for untested compounds limited the application of SAR study of flavonoids. However, the quantitative structure activity relationship (QSAR) for flavonoids as antibacterial agents is gaining interest^10^ by quantitatively correlating the molecular structures or properties with variation in biological activity.

3D-QSAR studies, mainly focus on how changes in 3D structural features such as electrostatic distribution, hydrophobic distribution^6^, hydrogen bond (H-bond) forming ability and orientation^11^ of chemical groups affect biological activity^12^. 3D-QSAR eliminates problems such as limitation in the prediction of stereochemistry of tested dataset and lack of recognition ability in search of active compounds^13^ suffered by the classical 2D-QSAR studies. Most commonly used methods in 3D-QSAR are comparative molecular field analysis^14^ (CoMFA) and comparative molecular similarity indices analysis^15^ (CoMSIA) since results are illustrated by visualized graphical contours which are easy to understand. CoMFA calculates steric and electrostatic fields while CoMSIA calculates five fields: steric, electrostatic, hydrophobic, H-bond donor and acceptor fields. CoMFA and CoMSIA have been successfully used to investigate the SARs of flavonoids binding site in GABA_A_ receptors^16^ and as inhibitors of PIM-1 kinase^17^ and breast cancer resistance protein^18^.

DNA gyrase is a type II DNA topoisomerase that introduces negative supercoils into DNA in an adenosine-triphosphate (ATP)-dependent reaction^19^. Gyrase exists only in prokaryotes making it an attractive target of antibacterial drugs^20^. Gyrase was found to contain two subunits GyrA and GyrB, with the A protein being responsible for DNA cleavage and rejoining, whereas the B protein contains the ATP-binding site and catalyzes ATP hydrolysis, providing the driving force for supercoiling of DNA^21^. A good correlation (*r* = 0.9582) between pIC_50_ values of flavonoids against purified *E. coli* DNA gyrase and MIC_50_ values of flavonoids against *E. coli* has been reported, suggesting that flavonoids may exert antibacterial activity by inhibiting the DNA gyrase^22^. Moreover, in docking studies, quercetin and epigallocatechin gallate (EGCG) have been reported to be potent GyrB inhibitors by competitively replacing ATP in the ATP binding pocket of GyrB^20^,^23^, resulting in the inhibition of DNA supercoiling activity. But the mode of action of flavonoids inhibits GyrB yet to be explained.

In this paper, molecular docking between 30 flavonoids and GyrB were performed to identify the key substituents and mode of action of flavonoids. To observe the effect of structure of flavonoids on their antibacterial activity, two 3D-QSAR models were developed by using two methods, CoMFA and CoMSIA. The main objectives of this study are to relate structure requirements of flavonoids to antibacterial activity and provide an explanation of the mechanism of flavonoids inhibiting GyrB. The chemical structures of the 30 flavonoids tested are presented in Table 1.

## Results

### Anti-*E. coli* activity of flavonoids

The tested flavonoids displayed varying levels of antibacterial activity against *E. coli* (Table 2). The IC_50_ values ranged from 25.21 μg/mL for **14** (flavonol) to 5290.09 μg/mL for **22** (flavanone). Other flavonols including **7** with IC_50_ of 35.76 μg/mL, **6** with IC_50_ of 53.49 μg/mL and **15** with IC_50_ of 141.79 μg/mL, also exhibited efficient inhibitory activities on *E. coli*. These results suggested that flavonols were more active than other flavonoid subclasses. Naringin **22**, showed the lowest antibacterial activity, indicating that glycosylation decreased antibacterial activity. Flavonoid glycosides **10**, **16** and **20** also showed lower activity than their corresponding flavonoid aglycons, **9**, **15** and **19**. Tangertin **1** showed higher activity than **2**, indicating that methoxylation at C-8 in the A ring had a positive effect on antibacterial activity. However, nobiletin **4**, with a methoxyl group at C-3′ in the B ring, showed lower activity than tangertin **1**, illustrating the methoxylation at this position decreased antibacterial activity.

### Validation of QSAR models

CoMFA and CoMSIA methods in SYBYL X-2.0^24^ were used to construct 3D-QSAR models to evaluate how change in 3D structural features of chemical substituent groups affects antibacterial activity. After omitting two outliers, dihydromyricetin **18** and formononetin **26**, a better *q*^*2*^ of 0.466 was obtained from a training set of 23 compounds as opposed to *q*^*2*^ of 0.237 derived from 25 compounds. ClogP (logarithm of the octanol/water partition coefficient) was used as an additional independent variable in 3D-QSAR models, leading to a higher *q*^*2*^ and prediction capability (Table 1s and Table 2s).

**Figure 1:**
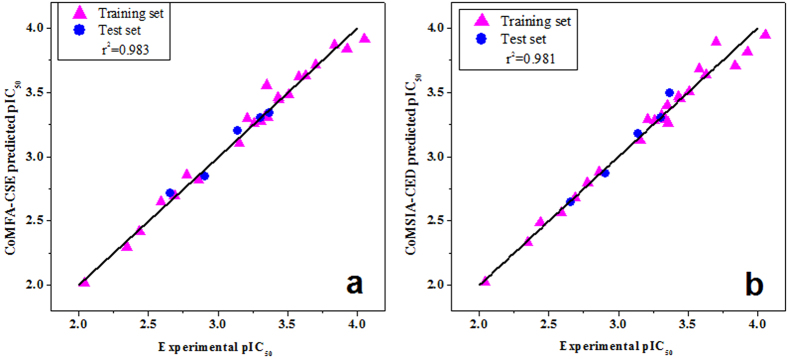
The predicted vs experimental pIC_50_ values for 23 compounds model. (**a**) CoMFA-CSE model; (**b**) CoMSIA-CED model. Filled magenta triangles (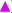) represent predictions for the training set; while filled blue circles (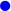) represent predictions for the test set.

The partial least squares (PLS) results of CoMFA-CSE and 31 possible CoMSIA-C field combinations are listed in Table 3. CoMSIA-CSE model with the highest *q*^*2*^ value was, however, not a good model for predictive capability due to its low correlation coefficient of 0.472 for prediction of the test set (Table 4 and Table 3s). CoMSIA-CED showed high correlation coefficient (*r*^*2*^ = 0.967) as well as high *q*^*2*^ (0.708). Supported by *r*^*2*^ value, this model had the ability to successfully predict compounds to eliminate in the training set. This model also satisfied all the other required parameters (Table 4) such as *r*_*m*_^2^, an indicator of good external predictability when its value is larger than 0.5^25^. For these reasons, CoMSIA-CED was chosen as the best CoMSIA model for further analysis. Figure 1 show the prediction curves obtained with final CoMFA-CSE and CoMSIA-CED models.

### CoMFA-CSE

The field contributions of CoMFA-CSE model of steric, electrostatic and ClogP descriptor were 35.6%, 45.7% and 18.7%, respectively (Table 3). The fact that contribution of ClogP was 18.7% and a good correlation (*r* = 0.66) obtained between ClogP and pIC_50_ indicates that hydrophobicity is important for antibacterial activities. Flavonoids need to penetrate the lipid bilayer cell membrane and interact with the membrane before reaching the target. Thus, the high ClogP is favorable for their antibacterial activity to some extent.

**Figure 2:**
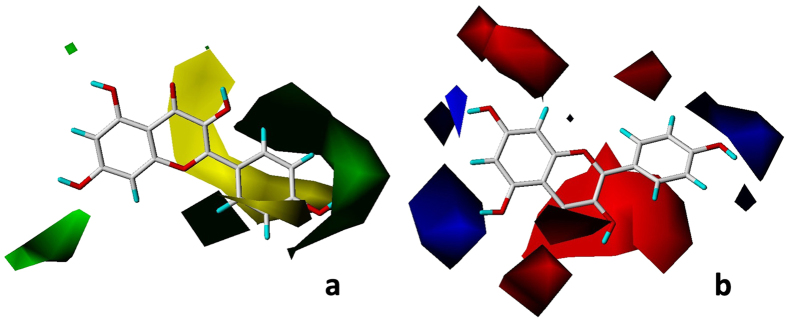
CoMFA-CSE contour maps based on kaempferol **14**, (**a**) steric field. Green contours indicate regions where bulk is favorable and yellow contours are areas where bulk is unfavorable; (**b**) electrostatic field. The blue region refers to the area where an electropositive group is favorable, while, the red region represents the area where an electronegative group is favorable.

In the CoMFA-CSE contour maps, steric field is illustrated in green (bulky favorable) and yellow (bulky unfavorable) contours (Fig. 2a). The steric favorable green contours near C-5 and C-7 in the A ring and C-2′ and C-4′ in the B ring indicated the importance of bulky groups in these areas for inhibitory activity. The presence of bulky substituent at C-5 increased the inhibitory activity in comparing **7** and **13**. One of steric unfavorable yellow contours was near C-3′ in the B ring (Fig. 2a), suggesting that a bulky group in this region decreased inhibitory activity as illustrated by the fact that pIC_50_ of **14** is higher than **7** and pIC_50_ of **1** is higher than **4**. The lower activity of **16** compared to **15** was supported by the yellow region near C-3, which was similar to **5** and **6**. A large yellow region under C-2, 3 in the C ring indicated that **7**, because of presence of unsaturated double bond, was much more active than **17**. The absence of an unsaturated double bond at C-2, 3 (planar structure) in flavonoids increases the spatial flexibility of the molecule by working like a bulky group.

In the CoMFA-CSE electrostatic contour map (Fig. 2b), red contours near C-7, C-8 in the A ring; C-3, C-4 in the C ring and C-2′, C-3′ and C-6′ in the B ring, indicated that electronegative groups around these areas increased the antibacterial activity of flavonoids. With an electronegative substituent (methoxyl group) at C-8, **1** showed higher activity than **2** (no substituent on the same position). Similar explanations are for the importance of electronegative group at C-3′ and C-3 by comparing **11** or **12** with **14**, respectively. pIC_50_ value (2.347) of **29**, a flavo-3-nol, was much lower than the pIC_50_ value (3.927) of **7**, implying the importance of carbonyl group at C-4. The blue areas near C-5, C-6 in the A ring and C-4′, C-5′ in the B ring illustrated the contribution of electropositive substituents. The antibacterial capacity was in the order **7** > **15**, suggesting that electronegative substituents at C-5′ decreased the inhibitory activity of flavonoids.

### CoMSIA-CED

**Figure 3:**
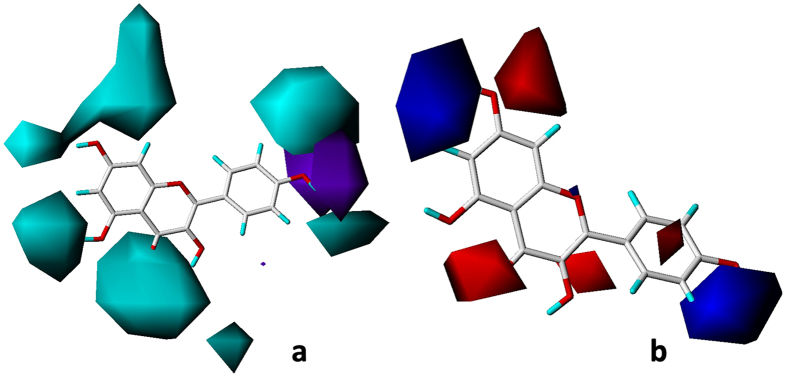
CoMSIA-CED contour maps based on kaempferol 14. (**a**) H-bond donor contour map, cyan contours indicate regions where an H-bond donor group is favorable and purple contours are areas where an H-bond acceptor group is favorable; (**b**) electrostatic field contour map, the blue region refers to the area where an electropositive group is favorable, while, the red region represents the area where an electronegative group is favorable.

The field contributions of CoMSIA-CED model of electrostatic, H-bond donor and ClogP descriptor were 34.8%, 44.8% and 20.4%, respectively (bold in Table 3).The contour map of the H-bond donor field in the CoMSIA-CED model is presented in Fig. 3a. Contours in cyan and purple depict the favorable and unfavorable H-bond donor groups, respectively. A large cyan contour around C-7 and C-8 suggested the presence of H-bond donor groups in this region would increase the antibacterial activity. Conversely, the presence of H-bond acceptor groups in this region would decrease the antibacterial activity as indicated by **8**, with a methoxyl group as the H-bond acceptor at C-8, having lower inhibitory activity than **5**. The cyan contours at C-5′ and C-3′ were supported by comparison of **17** versus **18** and **11** versus **12**, respectively. The cyan polyhedron in the vicinity of the hydroxyl groups at C-3 illustrated the enhancing effect on inhibitory activity of H-bond donor groups when comparing the SAR of **12** and **14.** However, opposite results that the presence of hydroxyl groups at C-3 was unfavorable for antibacterial activity were obtained by comparing **5** with **6**. This may be due to the strong electron attracting group of oxygen atom in hydroxyl group so that hydroxyl group can contribute both as an H-bond donor and H-bond acceptor. The only favorable H-bond acceptor group (purple contour) was located around C-4′ in the B ring (Fig. 3a).The hydroxyl group, acting as an H-bond acceptor resulted in an increase of antibacterial activity of **14** versus **6**.

The electrostatic field contour map of CoMSIA-CED model is illustrated in Fig. 3b. Red contours around C-3, C-4, C-7, C-8 and C-3′ suggest that electronegative substituents at these positions such as methoxyl or hydroxyl groups are important for inhibition against *E. coli*. Blue contours near C-6, C-7 and C-4′ indicate that electropositive substituents enhance the inhibitory activity. These results are consistent with the electrostatic field contour map of CoMFA-CSE model, confirming the importance of electrostatic field in flavonoids’ antibacterial activity.

### Molecular docking

Docking results of flavonoids and adenosine diphosphate (ADP) binding to GyrB are listed in Table 5. After docking, the conformation of the co-crystallized ligand ADP displayed the highest docking score (10.900) and CScore (5). Moreover, the root mean squared deviation (RMSD) value of the conformations of ADP was <2.0 Å between before and after docking (1.331 Å). The conformations and action modes of ADP are similar before and after docking (Fig. 1s).

**Figure 4:**
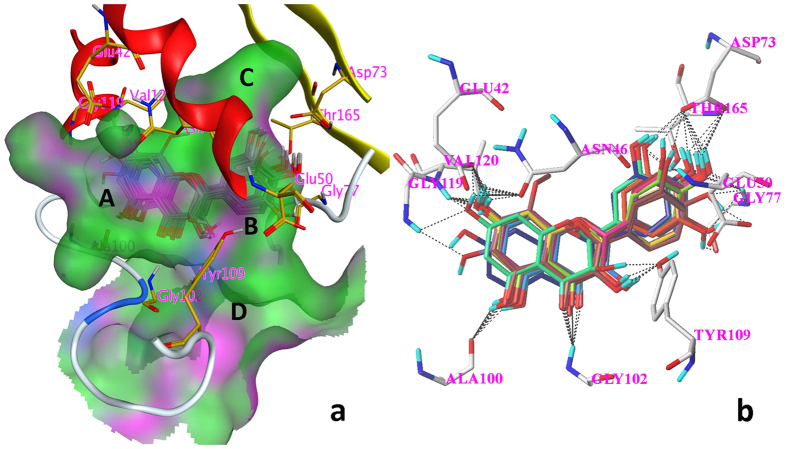
The predicted binding modes of 13 flavonoids to GyrB. Black dashed lines represent H-bonds between the flavonoids and the protein active site residues. (**a**) the interactions between 13 flavonoids and the surface of the pocket on GyrB (MOE); (**b**) the binding orientations of 13 flavonoids (SYBYL X-2.0) 13 flavonoids are **5**, **6**, **7**, **8**, **9**, **11**, **12**, **13**,**14**, **15**, **17**, **21** and **29**.

Same condition in the docking simulation of flavonoids was used based on the accurate reproduction of the binding mode of ADP. All of tested compounds can be generally considered as specific ligand of GyrB with high docking scores much more than 4 (Table 5). More than two-thirds of the tested compounds showed good CScores of 3 or more than 3 (Table 5). These results indicate that most of the tested flavonoids show good inhibitory activities to GyrB. Figure 4a shows binding modes of flavonoids to GyrB obtained by MOE^26^. A ring located at polar domain A formed by Glu42, Asn46, Gly119, Val120 on α helix; B ring placed at domains B, located in the center of the active site formed by Ala100, Gly102 and Tyr109 on coil; and C, formed by Gly77 on coil and Val71, Asp73 and Thr165 on β sheet; domain D occupying the opening of the pocket, contacting with solvent. The docking results illustrated that the orientations of 12 flavonoids are almost same to **14** including **5**, **6**, **7**, **8**, **9**, **11**, **12**, **13**, **15**, **17**, **21** and **29** (Fig. 4b). The top 5 antibacterial compounds (**5**, **6**, **7**, **9** and **11**) are also included in the common orientation of **14**, suggesting that the orientation of **14** is the probable active and common pose for the flavonoids whose molecular structure are similar to **14**.

**Figure 5:**
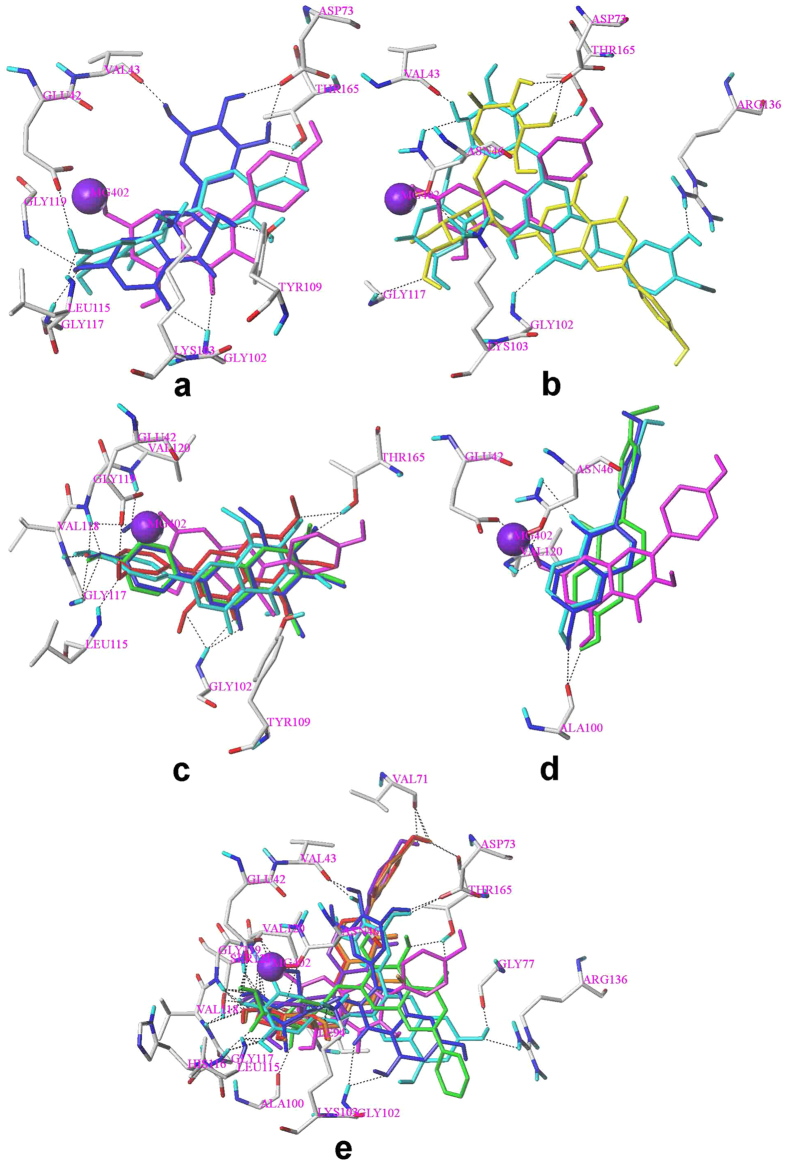
The predicted binding modes of 17 flavonoids to GyrB. Black dashed lines represent H-bonds between the flavonoids and the protein active site residues. Magnesium atom was shown in purple ball. The binding mode of compound **14** (magenta) was displayed for comparison. (**a**) **18**(blue) and **19** (cyan); (**b**) **20**(yellow) and **22**(cyan), two glycosidic groups superposed well to each other; (**c**) **1**(cyan), **2**(green), **3**(red) and **4**(blue); (**d**) **23**(cyan), **26**(green) and **27**(blue); (**e**) **10**(green), **16**(blue), **24**(red), **25**(purple), **28**(orange) and **30**(cyan), the glycosidic or gallate group superposed well to each other for all the flavonoids.

The orientations of the remaining 17 flavonoids are shown in Fig. 5. The poses for two flavanones **18** and **19** are similar to the pose of **14** (Fig. 5a). However, the other flavonoids did not match the pose of **14.** The orientation of all the flavonoid glycosides (**10**, **16**, **20**, **22**, **25**, **27** and **28**) and **30** (with a gallate group) are posed for superposing the glycosidic or gallate group near to magnesium atom (Fig. 5b,e). Moreover, three isoflavone glycosides **25**, **27** and **28** were well superposed to each other (Fig. 5e). The orientations of all the polymethoxyl flavones (**1**, **2**, **3** and **4**) were well superposed to each other but were not similar to **14** (Fig. 5c). The binding modes of isoflavone aglycons (**23**, **26** and **27**) were well superposed to each other as shown in Fig. 5d.

**Figure 6:**
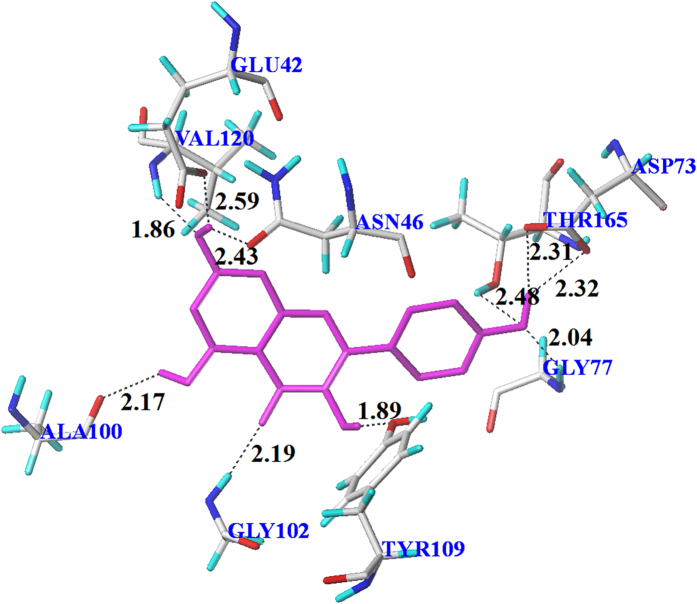
Interactions of **14** with key residues of GyrB (red, oxygen; blue, nitrogen; gray, carbon) in the binding cavity. Black dashed lines represent H-bonds and the numbers denote the distance of the H-bonds.

The substitution of hydroxyl groups formed multi-H-bonds with polar amino acid residues near the active site lead to the stable conformations of flavonoids (Fig. 4b). For simplicity, only the binding of kaempferol **14** to GyrB was analyzed in detail. Multiple H-bond (distances 1.86 to 2.59 Å) interactions formed between the surrounding amino acid residues and **14** (Fig. 6). The orientation of **14** in GyrB active site was stabilized by the strong H-bond network formed between two oxygen atoms of Thr165 and the hydrogen of 4′-hydroxyl, the oxygen atom of 4′-hydroxyl and hydrogen atoms of Asp73 and Gly77, respectively. This orientation was also stabilized by another H-bond network which formed between oxygen atoms of Glu42, Asn46 and the hydrogen atom of 7-hydroxyl, a hydrogen atom of Val120 and the oxygen atom of 7-hydroxyl. The hydrogen atoms of 3-hydroxyl and 5-hydroxyl were also involved in H-bond interactions with Tyr109 (1.89 Å) and Ala100 (2.17 Å), respectively. In addition, an H-bond (2.19 Å) was created between a hydrogen atom of Gly102 and the oxygen atom on 4-carbonyl. These binding modes provide an explanation for the high anti-*E. coli* activity of **14** and a common binding position of flavonoids for the inhibition of GyrB.

## Discussion

The results for antibacterial activity of flavonoids confirmed that flavonoids have potential antibacterial effects which were in agreement with the results of previous studies^8^,^10^,^27^. The fact that absence of hydroxyl group at C-3 decreased antimicrobial activity of flavonoids^28^ was in agreement with the fact that luteolin exhibited lower activity than quercetin. It was suggested that flavonols have higher antimicrobial effect than flavones^29^. Flavonoids aglycons showed higher antibacterial activity than corresponding flavonoid glycosides confirmed that glycosylation decreased antibacterial activity of flavonoids^9^. It is also important to note that additional information relating the structure requirements for flavonoids as antibacterial agents have been depicted in 3D-QSAR models than the limited results in the SAR studies.

The results obtained in both CoMFA-CSE and CoMSIA-CDE models were in agreement with previous studies, including the favorable negative charges at C-3^30^, C-7^27^, C-8 and C-2′^31^ and favorable positive charges at C-4′^22^. Moreover, bulky groups in CoMFA-CSE model at C-5^27^, C-7^32^, C-2′^33^, C-4′^33^ and C-5′^34^ were also consistent with the reported results. H-bond donor group (hydroxyl group) at C-5^22^ and C-7^27^ in CoMSIA-CED model could be beneficial for inhibition of *E. coli*. The 3D-QSAR models in this study propose that active positions including bulky unfavorable sites at C-4 and C-3′, electronegative groups at C-4 and C-3′, H-bond donor groups at C-3, C-4, C-8 and C-3′ and H-bond acceptor groups at C-4′ were also revealed in present study. In QSAR models, we focus on the change of structure of flavonoids related to antibacterial activity, but the mechanism for flavonoids’ antibacterial activity was not fully investigated.

GyrB has been proposed as the main binding target for antibacterial activity^35^. It had been reported that DNA supercoiling activity was inhibited by flavonoids’ overlapping in the ATP binding site of GyrB^20^,^25^. Molecular docking described an explanation for the mechanism for flavonoids’ antibacterial activities by illustrating the interaction between flavonoids and GyrB. 30 flavonoids were docked to the binding pocket, and 13 flavonoids showed the same orientation in the pocket (Fig. 4b). Most of these 13 flavonoids form H-bond or H-bond network with the Asp73 residue and an H-bond donor-H-bond acceptor pattern is essential for the formation of an H-bond network with Asp73 which is a common feature of the majority of GyrB inhibitors^36^,^37^,^38^. The hydroxyl group at C-4′ of flavonoids (**11**, **12**, **14**, **15**, **17**, **21** and **29**) formed H-bond or H-bond network with Gly77 and Tyr165 residues in the present study. Gly77, which would form H-bonds with inhibitor, has been identified as one of the essential amino acid residues in ATPase active site for the function of gyrase enzyme^38^,^39^,^40^. Thr165 forms H-bonds with ligand and is important for the successful inhibitor binding are confirmed in previous studies^37^,^38^,^41^. The top 6 antimicrobial compounds (**5**, **6**, **7**, **9**, **11** and **14**) formed an H-bond network to the side chain atoms of Asn46 and Glu42. Asn46 residue coordinates the Mg^2+^ in the ATP binding site and mutation at Asn46 was proved to abolish ATPase and supercoiling activities, suggesting the importance of this residue^38^,^39^,^42^, while, replacement of Glu42 by alanine reduced the activity of GyrB to undetectable levels^39^. Besides, H-bonds, formed from flavonoids with Gly102 and Ala100, are also important since flavonoid **13** (lack of H-bond formed between hydroxyl group at C-5 with Ala100) and **29** (lack of H-bond formed between carbonyl group with Gly102) showed low antibacterial activity.

The glycosylation played a very important role in the alteration of orientation of flavonoids as all the flavonoid glycosides changed their poses compared to the corresponding flavonoid aglycons. (Fig. 5). The multi-hydroxyl groups on glycosyl or gallate group of these compounds which can form numerous H-bonds with polar residues on GyrB may be responsible for the change of orientations of all the flavonoid glycosides and EGCG. Lose of H-bonds with crucial residues on GyrB of altered orientation of these flavonoids account for their low activity. For example, no H-bond was observed between **20** and **22** with Glu42 compared to **19** and **21**, respectively. The moderate activity of polymethoxyl flavones (**1**, **2**, **3** and **4**) could be explained as following: Substitution of three or more methoxyl groups increase the molecular volume and clash between polymethoxyl flavones and residues near the ATP pocket, resulting in different orientations and lose of the H-bonds with crucial residues (Fig. 5). Lack of H-bond donor group decreases the activity of polymethoxyl flavones since H-bond donor groups are necessary for the inhibitory activity as described in previous pharmacophore model^37^. However, methoxylation increase the hydrophobicity (Table 2) of polymethoxyl flavones result in an increase of hydrophobic interaction with surrounding residues^37^. Isoflavones **23**, **26** and **27** change their poses to avoid strong clash with Tyr109 located on the coil at domain D, which is flexible for the stretch and tight of the ATP pocket (Fig. 4a). This result was in agreement with CoMFA steric field that the bulky group at C-3 was unfavorable for antimicrobial activity. The alteration of orientations of isoflavones lost most of H-bonds between these flavonoids and crucial residues resulting in a decrease of inhibitory activity.

3-hydroxyl, 5-hydroxyl, 7-hydroxyl and 4-carbonyl were identified as key substituents for flavonoids as GyrB inhibitors. Similar results were reported for docking between GyrB and quercetin^20^. These key substructures were in accordance with the main active substituents of flavonoids identified in this QSAR study. In the QSAR study, calculations are based on the test compounds as ligands, where hydroxyl group normally functions as an H-bond donor with a polar hydrogen atom, resulting in many cyan contour as illustrated by around large area of kaempferol **14** (Fig. 3a). In molecular docking study, the calculations are based on the receptor of GyrB, hydroxyl group acting as either an H-bond donor or H-bond acceptor, forming intermolecular H-bond by binding to the corresponding H-bond acceptor or H-bond donor in the receptor. As shown in Fig. 6, the 4′-hydroxyl group acted as both H-bond donor (H) and acceptor (O).

In conclusion, the QSAR models demonstrated that hydrophobicity, H-bond donor, steric and electronic properties are key factors for the antibacterial activity of flavonoids. Structure requirements including hydroxyl group at C-3, C-5, C-7 and C-3′, C-2, 3 unsaturated double bond and the carbonyl group at C-4 are essential, while the presence of hydroxyl group at C-6, methoxyl group at C-8 and C-3′ could decrease the antibacterial activity. Docking results indicated that half of tested flavonoids inhibiting GyrB by interacting with ATP pocket in a same mode. Hydroxyl group at C-3, C-5, C-7 and C-4′, carbonyl group at C-4 are key substituents of flavonoids for inhibiting GyrB. Structure changes including glycosylation, polymethoxylation or isoflavonoids will change the action mode and result in a decrease of inhibitory activity. The findings of this study provide important information for efficiently screening of flavonoids with antibacterial activity. However, due to the limitation of the PLS analysis in CoMFA, more flavonoids need to be tested in both the training and test set in future to obtain better statistic model. Besides, more flavonoids also need to be tested in docking study to investigate a second preferred orientation for the flavonoids whose orientation is not similar to **14**.

## Materials and Methods

### Bacterial strain and chemicals

*E. coli* ATCC25922 was purchased from China Center of Industrial Culture Collection and used throughout the experiments. Compounds including **5**–**30** were purchased from Aladdin Chemistry Co., Ltd. (Shanghai, China) (purity > 98%, except **29**′s purity > 97%) while **1** and **4** were purchased from National Institute for Food and Drug Control (Beijing, China) (purity > 98%). **2** was purchased from ChromaDex Corp. (Irvine, CA) (purity > 98%). **3** was purchased from Yuan Mu Biotechnology Co., Ltd. (Shanghai, China) (purity > 98%). The flavonoids were dissolved in a small volume of dimethyl sulfoxide (DMSO), and the solutions were diluted with water to a final concentration of 1% DMSO. All other chemicals were of analytical grade.

### Antibacterial activity

The antibacterial activity was measured as IC_50_ which is defined as the concentration that inhibits the growth of 50% of organisms. The antibacterial activities of 10 compounds were measured in our lab previously^22^. The antibacterial activities of the remaining 20 compounds were measured by the same micro broth dilution method which was used to assess IC_50_ of each flavonoid performed in 96-well plate^22^. Briefly, two to three bacterial colonies were transferred into a tube containing 20 mL of Mueller-Hinton (MH) broth. After overnight incubation (37 °C and 150 rpm), concentration of bacteria was adjusted to 1 × 10^8^ colony forming units CFU/mL. Then bacteria were diluted with sterile MH broth (1 mL of bacteria/50 mL of MH). A 100 μL bacterial suspension containing 10^8^ cfu/mL of the bacteria (density matching the turbidity of a 0.5 McFarland standard) was added to each well. Then, 100 μL of each dissolved flavonoid stock was twofold serially diluted and transferred to each well with the test performed in a final volume of 200 μL. The same tests were performed for sterility control (MH broth) and growth control (MH broth + bacteria). The plates were covered and incubated for 16 h at 37 °C with shaking (220 rpm). The absorbance at 600 nm was measured using a microplate reader (Multiskan Spectrum, Thermo) to determine the bacterial growth. The inhibition ratio (%) was calculated as equation (1):


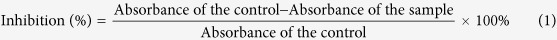


All experiments were performed in triplicate. Data analyses were performed by Origin 8.0 software.

### Conformational searching and molecular alignment

The IC_50_ values of 30 compounds were converted to pIC_50_ values. The test set, comprising 5 compounds (**8, 12, 13, 20** and **27**) selected at random, was used to evaluate the predictive power of the resulting models (Table 1). The remaining 25 compounds were used as independent variables in the CoMFA and CoMSIA analyses (Table 2). Each structure of 30 flavonoids was drawn using the sketch function in SYBYL-X 2.0. The method of energy minimization was Powell. Force field and charges were set in Tripos and Gast-Hück, respectively. Max iteration was 1000 and termination was a convergence criterion 0.005 kcal/ (mol*Å). Other parameters were established using default values.

**Figure 7:**
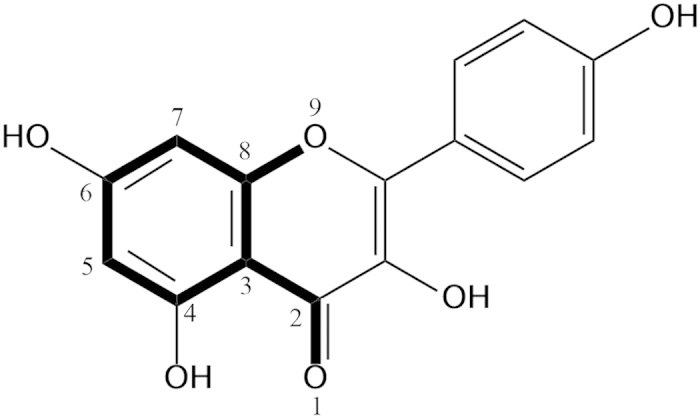
kaempferol 14 was used as template for molecular alignment. The common structure, showing in bold face and numbered 1–9 is used in alignment database of the SYBYL X-2.0 program.

In CoMFA and CoMSIA studies, 3D structures of the molecules are required to be aligned based on a suitable conformational template and its substructure, which is assumed to be a “bioactive” conformation^43^. Compound with the highest pIC_50_ value was used as the template for molecular alignment. Figure 7 shows common substructure in bold lines (atoms from No. 1 to 9). 23 compounds of training set were aligned and well-aligned database was obtained by using the SYBYL align database methods. The remaining **29** and **30**, without carbonyl group at C-4 in the C ring (Table 1), were aligned with a common structure (atoms from No. 2 to 9) by using the SYBYL match methods. The 5 compounds in test set were also aligned based on the template kaempferol **14** and saved as another database.

### CoMFA

CoMFA is an alignment-dependent descriptor method. Molecular field interaction energies are calculated and correlated with biological activities/responses by using multivariate statistical analyses^44^. Two compounds, **18** and **26** was omitted as outliers at the beginning of modeling due to low biological activity and structure uniqueness^43^. The training set of 23 molecules which had been aligned were placed in a rectangular grid, the probe of *sp*^*3*^ carbon with +1 net charge was used to calculate the steric and electrostatic fields energies and the cutoff was set at 30 kcal/mol. The pIC_50_ values of flavonoids were used as the dependent variables, CoMFA values as independent variable. A better of *q*^*2*^ was obtained when ClogP was used as an additional independent variable. Regression analyses of these variables were performed using the PLS algorithms with default parameters. To acquire the optimal principal components (PCs) and *q*^*2*^ value for PLS procedure, the cross-validation analyses were performed by using leave-one-out (LOO) with 6 principal components^45^. Sample-distance partial least squares (SAMPLS) method was used to increase the calculation speed. This option vastly increases the speed with which cross validation results are returned^46^. The no-validation analyses were performed by using PLS with the number of optimal components corresponding to the optimal *q*^*2*^ value. Both of the predicted pIC_50_ in training set and test set were obtained from the CoMFA model.

### CoMSIA

CoMSIA is a modified method of CoMFA^44^. In CoMSIA, The calculation function used in the CoMSIA analyses is Gaussian-type distance which avoid of singularities occurring at atom positions. Thus, no arbitrary cutoffs were required^34^. CoMSIA similarity indices(A_F_) were as described in equation (2)^43^:


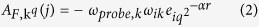


Five types of similarity index fields (*k*) were calculated for the well-aligned molecules, including steric, electrostatic, hydrophobic, H-bond donor and acceptor. The SYBYL default attenuation factor value (α) of 0.3 was used, which illustrated the steepness of the Gaussian-type function. The pIC_50_ values of all flavonoids in training set were used as the dependent variables, one or more *k* values as independent variables. A better of *q*^*2*^ was obtained when ClogP was used as an additional independent variable. Regression analyses of these variables were performed as CoMFA operation. 31 models are available in CoMSIA model construction. The predicted pIC_50_ for both training set and test set were obtained from the best CoMSIA model.

### Validation of QSAR models

Though, a high value of *q*^2^ is important, it alone is not sufficient for a predictive model. To determine the best CoMSIA model, another evaluation was made by calculating the additional parameters of CoMFA and five CoMSIA models which shared high *q*^*2*^ values. Linear regressions were analyzed for the observed and predicted values of the test set compounds with intercept (squared correlation coefficient *r*^*2*^, slope *k*) and without intercept (squared correlation coefficient *r*_*0*_^*2*^, slope *k’*)^47^. Models are considered acceptable if they meet equations (3) and (4) and all of the following conditions: 0.85 ≤ *k* ≤ 1.15 or 0.85 ≤ *k*′ ≤ 1.15; *q*^*2*^ > 0.5 and *r*^*2*^ > 0.6.


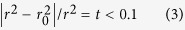



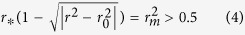


All the above parameters are calculated to evaluate predictive ability of the obtained QSAR models by CoMFA and CoMSIA^48^.

### Molecular docking

The antibacterial target GyrB was selected as the target receptor for molecular docking study. Surflex-Dock^49^ (SFXC) algorithm was used to dock the co-crystalized ligand ADP and compounds to the active site GyrB (PDB ID: 4PRV) of *E. coli*. ADP was removed from the structure, water molecules were removed and essential hydrogen atoms were added. Gast-Hück charges were calculated for the ligand, while AMBER 7 FF99 charges were used for the protein. The model was then subjected to energy minimization. A *protomol* was generated by the mode of ligand. Essential hydrogen atoms were added for ligands. The additional starting conformations per molecule were set to 20. Other parameters were established using default values. Firstly, ADP extracted from the GyrB was docked back to the active site on GyrB to validate the rationality of parameters set in docking process by comparing the conformations and RMSD value of the Co-crystalized ADP orientation and ADP orientation after docking. Given two sets of npoints V and W, the RMSD value was calculated as shown in equation (5). Where δ is the distance between N pairs of equivalent atoms. Then, the database comprised by 30 flavonoids was docked to the active site.


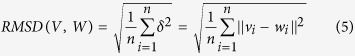


Docking score is expressed in p*K*_*d*_ represents binding affinities. A compound will be generally considered to be a specific ligand of a protein when its docking score is greater than 4.0^50^. Consensus scores (CScore), combining several popular scoring functions for ranking the binding affinity of ligands to a receptor, ranges from 1 to 5. The best CScore according to the literature is 5^49^. Crash represents the degree of inappropriate penetration into the protein by the ligand as well as the degree of internal self-clashing that the ligand is experiencing. Crash values that are close to 0.0 are favorable.

## Additional Information

**How to cite this article**: Fang, Y. *et al*. 3D-QSAR and docking studies of flavonoids as potent *Escherichia coli* inhibitors. *Sci. Rep*. **6**, 23634; doi: 10.1038/srep23634 (2016).

## Supplementary Material

Supplementary Information

## Figures and Tables

**Table 1:**
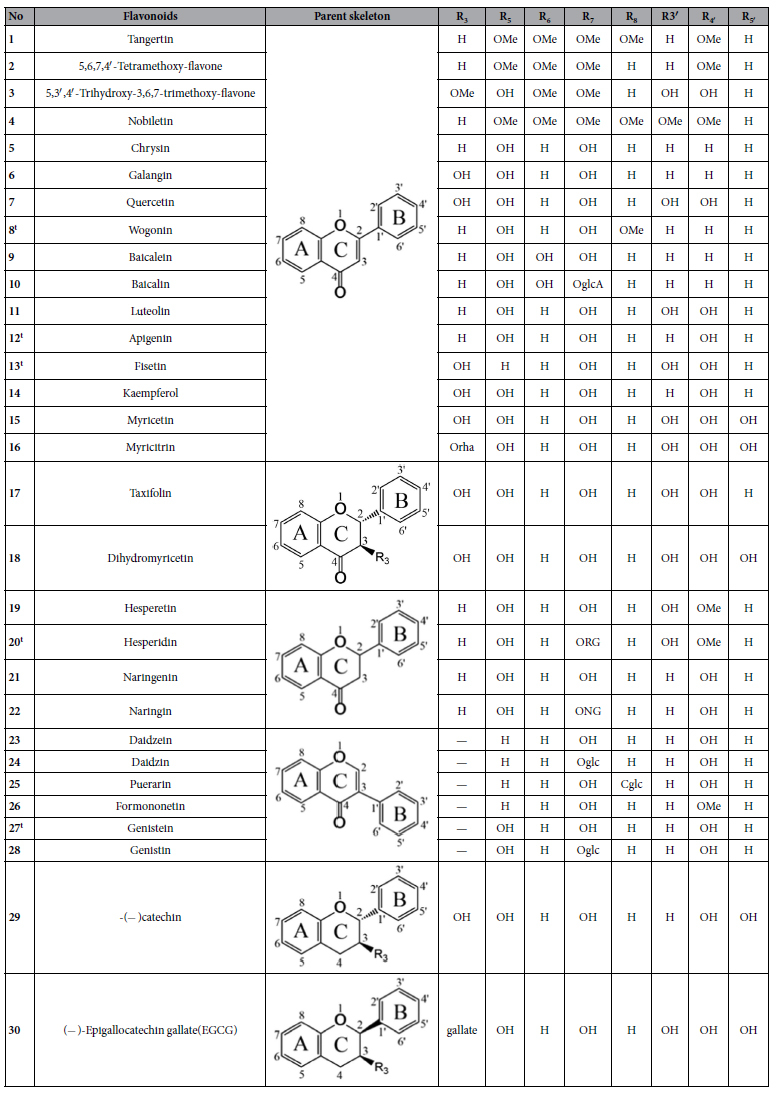
The chemical structures of 30 flavonoids.

Cglc: -C-glucopyranosyl; Oglc: -O-glucopyranosyl; OglcA: -O- glucuronyl; Orha: -O-α-L-rhamnopyranosyl; RG: -(6-O-(6-deoxy-α-L- mannopyranosyl)-β-D-glucopyranosyloxy); NG: -(2-O-(6-deoxy-α-L-mannopyranosyl)- β-D- glucopyranosyloxy); t: Test set.

**Table 2:** IC_50_, pIC_50_, ClogP values and molecular weight (MW) of 30 flavonoids.

No	IC_50_(μg/mL)	pIC_50_	ClogP	MW	No	IC_50_ (μg/mL)	pIC_50_	ClogP	MW
**1**	137.12^22^	3.434	2.39	372.37	**16**	953.20	2.688	−0.45	464.38
**2**	156.25^22^	3.341	2.80	342.34	**17**	781.25	2.590	0.77	304.25
**3**	497.56	2.860	1.66	360.31	**18**	119.41	3.428	0.10	320.25
**4**	177.05^22^	3.357	2.12	402.40	**19**	93.75	3.508	2.29	302.28
**5**	36.72^22^	3.840	3.56	254.24	**20**	762.10	2.904	−0.29	610.56
**6**	53.49^22^	3.703	2.76	270.24	**21**	167.48	3.211	2.44	272.25
**7**	35.76^22^	3.927	1.50	302.24	**22**	5290.09	2.040	−0.09	580.54
**8**	121.23	3.370	3.33	284.26	**23**	124.52	3.310	2.08	254.24
**9**	70.94^22^	3.581	3.00	270.24	**24**	151.06	3.440	0.37	416.38
**10**	312.50	3.155	0.77	446.36	**25**	1521.37	2.437	0.02	416.38
**11**	67.25^22^	3.629	2.31	286.24	**26**	712.50	2.576	2.64	268.26
**12**	195.43	3.141	2.91	270.24	**27**	248.20	3.307	2.41	270.24
**13**	625.82	2.660	1.24	286.24	**28**	238.23	3.259	0.91	432.38
**14**	25.21^22^	4.055	2.10	286.24	**29**	1304.58	2.347	0.53	290.27
**15**	141.79^22^	3.351	0.84	318.24	**30**	764.61	2.778	1.49	458.37

^22^Data derived from reference 22.

**Table 3:** Partial least square (PLS) analysis of CoMFA and CoMSIA models.

No	N	*q*^*2*^	SEE	*R*^*2*^	*F*	Contribution					
						C	S	E	H	D	A
CoMFA-CSE	5	0.743	0.077	0.983	210.147	0.187	0.356	0.457			
CoMSIA-C											
S	6	0.528	0.185	0.910	27.085	0.118	0.882				
E	4	0.699	0.114	0.962	113.297	0.232		0.768			
H	3	0.478	0.202	0.873	43.46	0.257			0.743		
D	6	0.569	0.093	0.978	116.035	0.184				0.816	
A	5	0.499	0.170	0.920	38.875	0.251					0.749
SE	6	0.72	0.079	0.984	159.739	0.195	0.200	0.605			
SH	3	0.482	0.198	0.878	45.611	0.263	0.191		0.546		
SD	6	0.556	0.086	0.981	134.625	0.185	0.171			0.644	
SA	6	0.563	0.129	0.956	58.232	0.204	0.238				0.558
EH	5	0.706	0.083	0.981	174.887	0.199		0.412	0.389		
**ED**	**6**	**0.708**	**0.072**	**0.986**	**194.226**	**0.204**		**0.348**		**0.448**	
EA	5	0.624	0.118	0.961	84.871	0.225		0.455			0.321
HD	4	0.55	0.118	0.959	104.807	0.196			0.359	0.445	
HA	4	0.602	0.135	0.947	79.957	0.232			0.45		0.318
DA	6	0.585	0.106	0.971	88.431	0.21				0.499	0.291
SEH	6	0.705	0.069	0.988	214.624	0.193	0.107	0.359	0.341		
SED	6	0.711	0.073	0.986	186.529	0.203	0.095	0.314		0.388	
SEA	6	0.646	0.098	0.975	102.655	0.203	0.118	0.408			0.271
SHD	4	0.546	0.126	0.953	92.124	0.201	0.098		0.318	0.383	
SHA	4	0.569	0.134	0.947	81.024	0.217	0.112		0.404		0.267
SDA	6	0.594	0.102	0.973	95.869	0.207	0.106			0.441	0.246
EHD	6	0.686	0.061	0.990	271.105	0.178		0.268	0.248	0.306	
EHA	6	0.68	0.077	0.985	171.291	0.202		0.298	0.300		0.201
EDA	6	0.669	0.087	0.980	131.369	0.211		0.266		0.338	0.185
HAD	4	0.605	0.118	0.959	104.588	0.217			0.275	0.318	0.190
SEHD	6	0.682	0.070	0.987	208.212	0.182	0.067	0.247	0.228	0.277	
SEHA	6	0.67	0.080	0.983	155.882	0.197	0.074	0.274	0.276		0.179
SEDA	6	0.671	0.087	0.98	131.428	0.208	0.068	0.248		0.310	0.166
SHDA	4	0.59	0.119	0.958	102.718	0.212	0.068		0.257	0.294	0.168
EHDA	6	0.678	0.074	0.986	183.235	0.190		0.217	0.207	0.244	0.142
SEHDA	6	0.666	0.076	0.985	173.278	0.189	0.053	0.203	0.196	0.229	0.130

*q*^*2*^: Cross-validated correlation coefficient after the leave-one-out procedure. N: Optimum number of components, *R*^*2*^: Non-cross-validated correlation coefficient, SEE: standard error of estimate, *F*: F-test value. C, ClogP; S, steric; E, electrostatic; H, hydrophobic; D, H-bond donor; A, H-bond acceptor. CoMSIA-CED model was chosen as final CoMSIA model which was shown in bold.

**Table 4:** Validation for predictive ability of QSAR models.

Models	*q*^*2*^	*r*^*2*^	*k*	*k'*	*r*_*m*_^2^	*t*
CoMFA-CSE	0.743	0.966	1.001	0.943	0.948	0.004
CoMSIA-CSE	0.72	0.472	0.972	1.092	0.436	0.013
CoMSIA-CED	0.708	0.967	1.007	1.142	0.945	0.014
CoMSIA-CSED	0.711	0.682	1.015	1.263	0.572	0.041
CoMSIA-CSEH	0.705	0.445	0.986	0.867	0.403	0.020
CoMSIA-CSEHDA	0.666	0.67	1.017	1.067	0.622	0.007

C, ClogP; S, steric; E, electrostatic; H, hydrophobic; D, H-bond donor; A, H-bond acceptor; linear regressions with intercept (squared correlation coefficient *r*^*2*^, slope *k*) and without intercept (squared correlation coefficient *r*_*0*_^*2*^, slope *k′* ).

**Table 5:** Docking results of 30 flavonoids and co-crystalized ligand (ADP).

Name	DockingScore	Crash	Polar	CScore	Name	DockingScore	Crash	Polar	CScore
ADP	10.90	−2.43	9.71	5	**16**	10.72	−1.72	7.51	4
**1**	8.01	−2.51	2.05	3	**17**	7.57	−1.96	7.02	3
**2**	7.73	−1.81	3.18	3	**18**	7.71	−1.12	5.36	2
**3**	8.16	−2.47	4.87	2	**19**	7.72	−2.05	4.99	1
**4**	9.31	−2.86	4.18	3	**20**	7.90	−6.22	5.07	4
**5**	6.20	−0.70	3.64	3	**21**	8.00	-1.13	6.00	3
**6**	7.19	−0.67	4.70	2	**22**	8.30	−4.61	4.74	5
**7**	7.14	−1.03	5.40	3	**23**	5.85	−0.64	0.74	2
**8**	6.78	−0.87	3.52	2	**24**	8.97	−4.39	4.09	2
**9**	6.39	−0.58	3.52	3	**25**	7.78	−4.68	5.29	4
**10**	9.72	−1.93	5.75	2	**26**	7.19	−1.09	0.81	4
**11**	7.62	−1.53	6.34	4	**27**	5.97	−0.92	2.45	2
**12**	7.52	−1.10	5.21	3	**28**	8.25	−3.95	3.66	4
**13**	7.14	−1.39	4.41	3	**29**	8.10	−1.26	5.93	5
**14**	8.31	−1.15	6.43	4	**30**	10.68	−2.07	6.98	5
**15**	8.60	−1.71	7.55	4					
