# Supplementary material for: 3D-QSAR and docking studies of flavonoids as potent Escherichia coli inhibitors
Source: Sci Rep. 2016 Apr 6;6:23634. doi: 10.1038/srep23634 (PMC4822154; doi:10.1038/srep23634)
Supplement: Supplementary Information [file srep23634-s1.doc]

**3D-QSAR and docking studies of flavonoids as potent *Escherichia coli* inhibitors**

Yajing Fang^1^, Yulin Lu^2^, Xixi Zang^3^, Ting Wu^1^, XiaoJuan Qi^4^, Siyi Pan^1^ & Xiaoyun Xu^1*^

At the beginning of modeling, 25 compounds as training set with or without ClogP descriptor, 23 compounds as training set with or without ClogP descriptor are taken into account to find the best model. The PLS statistics results are listed in Table 1s.

Table 1s PLS statistics of CoMFA and CoMSIA 3D QSAR models

| PLS  statistics | 25 compounds model | | | | | | | 23 compounds model | | | | | | |
| --- | --- | --- | --- | --- | --- | --- | --- | --- | --- | --- | --- | --- | --- | --- |
|  | Without ClogP | |  | | With ClogP | |  | | Without ClogP | |  | | With ClogP | |
|  | CoMFA | CoMSIA | | CoMFA | | CoMSIA | | CoMFA | | CoMSIA | | CoMFA | | CoMSIA |
| *q^2^* | 0.237 | 0.013 | | 0.376 | | 0.222 | | 0.466 | | 0.509 | | 0.743 | | 0.666 |
| SEE | 0.214 | 0.259 | | 0.179 | | 0.390 | | 0.102 | | 0.071 | | 0.077 | | 0.076 |
| *R^2^* | 0.847 | 0.775 | | 0.898 | | 0.467 | | 0.971 | | 0.987 | | 0.983 | | 0.985 |
| N | 2 | 2 | | 3 | | 1 | | 5 | | 6 | | 5 | | 6 |
| *F* | 60.784 | 37.886 | | 61.438 | | 20.163 | | 113.618 | | 201.004 | | 201.147 | | 173.278 |
| Contribution | | | | | | | | | | | | | | |
| C | - | - | | 0.116 | | 0.487 | | - | | - | | 0.187 | | 0.189 |
| S | 0.448 | 0.086 | | 0.373 | | 0.051 | | 0.462 | | 0.071 | | 0.356 | | 0.053 |
| E | 0.552 | 0.212 | | 0.511 | | 0.093 | | 0.538 | | 0.223 | | 0.457 | | 0.203 |
| H | - | 0.236 | |  | | 0.125 | |  | | 0.248 | |  | | 0.196 |
| D | - | 0.258 | |  | | 0.118 | |  | | 0.283 | |  | | 0.229 |
| A | - | 0.208 | |  | | 0.126 | |  | | 0.175 | |  | | 0.130 |

*q^2^*: Cross-validated correlation coefficient after the leave-one-out procedure; SEE: standard error of estimate; *R^2^*: Non-cross-validated correlation coefficient; N: Optimum number of components; *F*: *F*-test value. C: ClogP; S: steric; E: electrostatic; H: hydrophobic; D: H-bond donor; A: H-bond acceptor.

The residuals obtained from different models of 7 compounds including the test set (**8**, **12**, **13**, **20** and **27**) and outliers (**18** and **26**) were shown in Table 2s.

Table 2s Residuals of the predictions of 7 flavonoids by the CoMFA and CoMSIA models

| No. | pIC_50_ | Residuals | | | | | | | | | | | | | |
| --- | --- | --- | --- | --- | --- | --- | --- | --- | --- | --- | --- | --- | --- | --- | --- |
|  |  | 25 compounds model | | | | | | | 23 compounds model | | | | | | |
|  |  | Without ClogP | |  | | With ClogP | |  | | Without ClogP | |  | | With ClogP | |
|  |  | CoMFA | CoMSIA | | CoMFA | | CoMSIA | | CoMFA | | CoMSIA | | CoMFA | | CoMSIA |
| **8** | 3.370 | 0.255 | -0.305 | | -0.469 | | -0.347 | | 0.263 | | 0.321 | | 0.028 | | -0.160 |
| **12** | 3.141 | 0.722 | -0.518 | | -0.827 | | -0.479 | | 0.809 | | 0.71 | | -0.057 | | -0.193 |
| **13** | 2.660 | 0.947 | -0.814 | | -0.859 | | -0.547 | | 1.093 | | 1.011 | | -0.058 | | -0.218 |
| **20** | 2.904 | 0.167 | -0.300 | | 0.025 | | 0.185 | | -0.07 | | 0.016 | | 0.057 | | 0.309 |
| **27** | 3.307 | 0.284 | -0.019 | | 0.340 | | -0.110 | | -0.091 | | 0.19 | | 0.003 | | -0.006 |
| **18** | 3.428 | -0.276 | -0.476 | | -0.31 | | -0.608 | | -0.494 | | -0.93 | | -0.795 | | -1.024 |
| **26** | 2.576 | 0.399 | 0.651 | | 0.244 | | 0.253 | | 0.828 | | 0.961 | | 1.063 | | 1.016 |

The predicted pIC_50_ of test set obtained from the best CoMFA and 6 CoMSIA models which share high *q^2^* values are revealed in Table 3s. These values were used to validate the predictive ability of QSAR models

Table 3s Experimental vs predicted pIC_50_ of the test set by the CoMFA and CoMSIA models

| Name | Experimental  pIC_50_ | Predicted pIC_50_ | | | | | | |
| --- | --- | --- | --- | --- | --- | --- | --- | --- |
|  |  | CoMFA-C |  | CoMSIA-C | | | | |
|  |  | SE |  | SE | ED | SED | SEH | SEDHA |
| **8** | 3.370 | 3.342 |  | 3.350 | 3.491 | 3.596 | 3.340 | 3.530 |
| **12** | 3.141 | 3.198 |  | 3.204 | 3.176 | 3.369 | 3.205 | 3.334 |
| **13** | 2.660 | 2.718 |  | 2.855 | 2.643 | 2.789 | 2.947 | 2.878 |
| **20** | 2.904 | 2.847 |  | 2.233 | 2.868 | 2.531 | 2.415 | 2.595 |
| **27** | 3.307 | 3.304 |  | 3.303 | 3.299 | 3.305 | 3.279 | 3.313 |

S: steric; E: electrostatic; H: hydrophobic; D: H-bond donor; A: H-bond acceptor; CoMFA-C: CoMFA-ClogP; CoMSIA-C: CoMSIA-ClogP.

ADP extracted from the GyrB was docked back to the active site on GyrB to validate the rationality of parameters set in docking process. The conformations and action modes of ADP before and after docking are similar as shown in Figure 1s.


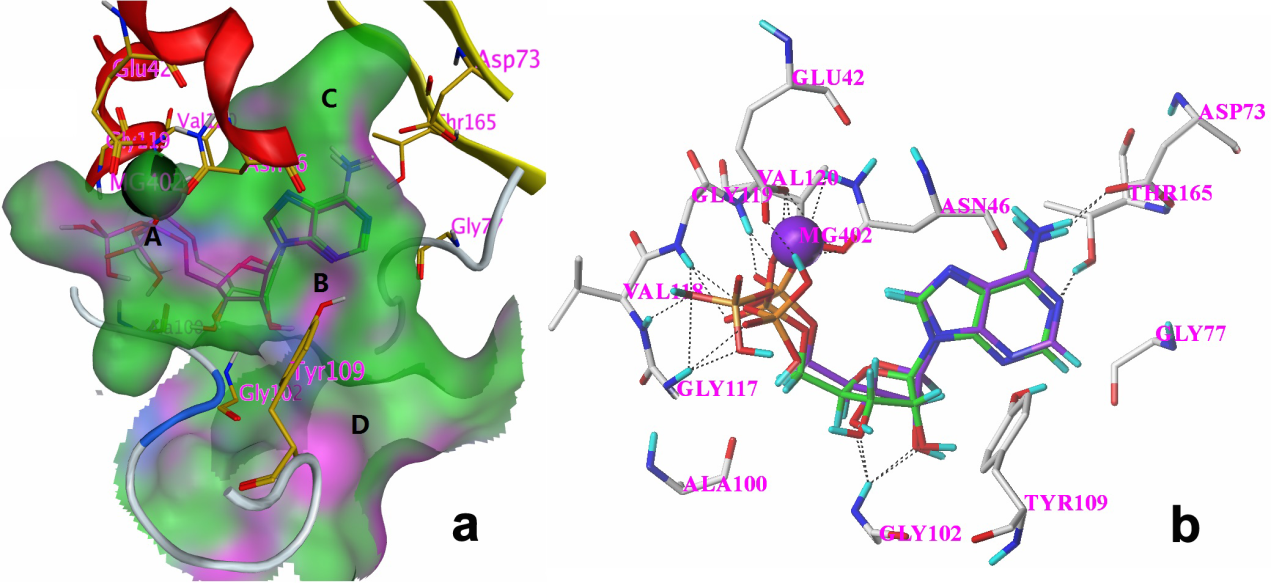


Figure1s. The binding orientations of superimposed ADPs before (purple) and after (blue) docking. **a**: the interactions between ADPs and the surface of the pocket on GyrB (MOE); **b**: the binding orientations of ADPs (SYBYL X-2.0).
